# The Role of Omega-3 Polyunsaturated Fatty Acids in Patients with Metabolic Syndrome and Endothelial Dysfunction

**DOI:** 10.3390/medicina61010043

**Published:** 2024-12-30

**Authors:** Andrej Dzupina, Dominik Pella, Pavol Zenuch, Zuzana Zenuchova, Jaipaul Singh, Monika Jankajova, Jan Fedacko

**Affiliations:** 1 Department of Angiology, National Heart Institute, 040 11 Bratislava, Slovakia; a.dzupina@gmail.com; 2 Centre of Clnical and Preclinical Research, MEDIPARK–University Research Park, Pavol Jozef Safarik University, Trieda SNP 1, 040 11 Kosice, Slovakia; dominik.pella@gmail.com (D.P.); pavolzenuch@gmail.com (P.Z.); zenuchovazuzka@gmail.com (Z.Z.); jan.fedacko@upjs.sk (J.F.); 3 School of Pharmacy and Biomedical Sciences, University of Central Lancashire, Preston PR1 2HE, UK; jaipsingh10@gmail.com; 4 Kardiocentrum AGEL SACA, Lúčna 57/040 15, 040 15 Košice, Slovakia; 5 SLOVACRIN, Slovak Clinical Research Infrastructure Network, Faculty of Medicine, Pavol Jozef Safarik University, Trieda SNP 1, 040 11 Kosice, Slovakia

**Keywords:** metabolic syndrome, endothelial dysfunction, heart diseases, omega-3 fatty acids

## Abstract

*Background and Objectives:* Metabolic syndrome (MS) represents several diseases encompassing a heterogeneous group of biochemical and physiological abnormalities characterized by structural and functional alterations in the myocardium, including the endothelium of the coronary arteries. MS also affects a substantial portion of the global population. Understanding the risk factors, the development and treatment associated with MS are of paramount importance for early identification, treatment and prevention. This study was designed to evaluate the role of the supplementation of omega-3 polyunsaturated fatty acids (n-3 PUFAs) on endothelial function in patients with MS. *Materials and Methods:* A total of 80 patients with MS were enrolled in two groups. The study evaluated endothelial function (EF) in subjects before and after a three-month treatment with n-3 PUFAs in a dose of 2.4 g daily (800 mg, three times a day) vs. placebo, using an Endo-PAT2000 device (Itamar Medical Ltd., Caesarea, Israel) measuring the reactive hyperemia index (a parameter of EF) and augmentation index (a parameter of arterial stiffness). Plasmatic levels of glutathione peroxidase, homocysteine, apolipoprotein B and lipoprotein were also evaluated for comparison. *Results:* The results showed that the average value of reactive hyperemia index before the treatment with n-3 PUFAs was 1.62 ± 0.42, compared to 1.96 ± 0.62 at the end of the study (*p* < 0.005). The augmentation index changed from 14.66 ± 19.55 to 9.21 ± 15.64 after the treatment (*p* = 0.003) with n-3 PUFA. The results also revealed a statistically significant decrease in apolipoprotein B (0.94 ± 0.36 vs. 1.13 ± 0.35, *p* = 0.001) and homocysteine (19.31 ± 5.29 vs. 13.78 ± 3.05, *p* = 0.001) and an increase in glutathione peroxidase plasma levels (41.65 ± 8.90 vs. 45.20 ± 8.01), *p* = 0.001. *Conclusions:* The results of this prospective study showed a significant improvement in EF in subjects with MS treated with n-3 PUFAs in a dose of 2.4 g daily.

## 1. Background

Despite advances in preventive cardiology, cardiovascular (CV) mortality remains high globally. Metabolic syndrome (MS) is defined as a simultaneous presence of lipid- and non-lipid-related CV and cardio-metabolic risk factors that significantly increase the risk of CV disorders, as well as type 2 diabetes (T2DM). A common trait of these risk factors is the fact that they cause endothelial dysfunction (ED). ED plays a significant role in the development and clinical manifestation of atherosclerosis [1].

Omega-3 poly-unsaturated fatty acids (n-3 PUFAs) used for the primary and secondary prevention of CV disorders are currently associated with a wide range of evidence within the framework of evidence-based medicine. Their anti-inflammatory, anti-thrombotic, anti-arrhythmic and triglyceride-reducing effects (at high doses) are well established and often associated with an increase in HDL cholesterol. The objective of this work was to assess the possible effect of n-3 PUFA on EF in MS patients [2].

The first evidence of the benefits of n-3 PUFA originated in 1970s, based on published reports revealing a reduction in the prevalence of ischemic heart disease (IHD) and diabetes in Eskimos Inuit’s, who lived in Greenland and who were found to have high n-3 PUFA levels due to an excessive intake of fish fat that contained n-3 PUFA [1]. These reports have been expanded over recent decades, extended, supplemented and analyzed in additional clinical studies. The results of GISSI Prevenzione and DART studies present an explanation for several effects of n-3 PUFA [2,3].

Clinical data supporting a reduction in the progression of atherosclerosis with n-3 PUFA originated from SCIMO (Study on the Prevention of Coronary Atherosclerosis by Intervention with Marine Omega-3 fatty acids). This study undertook a quantitative analysis of the progression and regression of plaque in coronary arteries [4]. Based on extensive evidence-based medicine data in the primary and secondary prevention of CV diseases, n-3 PUFAs have their role in the recommendations of international and global organizations [5].

Considering the results from previous studies, the current study was designed to evaluate the role of n-3 PUFA supplementation on EF in patients with MS compared to a placebo group.

## 2. Methodology

### 2.1. Study Population

A total of 80 patients with MS out of 984 (screened) were enrolled in the study (40 patients on n-3 PUFAs and 40 patients on a corresponding placebo). Patients were randomly divided among study subgroups, and the investigators were blinded to the investigating product.

The patient population was based on the number of patients examined at the out-patient cardiology office (academic-based) of Pavol Jozef Safarik University in Kosice, as well as the patients referred by cooperating cardiologists. The main exclusion criteria were MS and patients who took any nutrition supplements containing n-3 PUFAs in the last 6 months. Other exclusion criteria were a known hypersensitivity to the study treatments and conditions that in the opinion of the investigator would be associated with poor adherence to the protocol. When enrolling the patients, the trial focused especially on clinically stable patients who were not expected to have either their pharmacotherapy or lifestyle changing during the observed study period.

**Figure 1:**
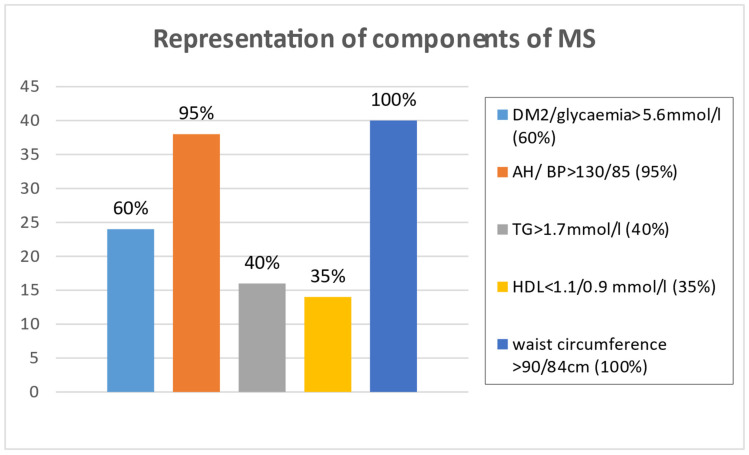
Representation of components of metabolic syndrome in n-3 PUFA group of patients.

**Figure 2:**
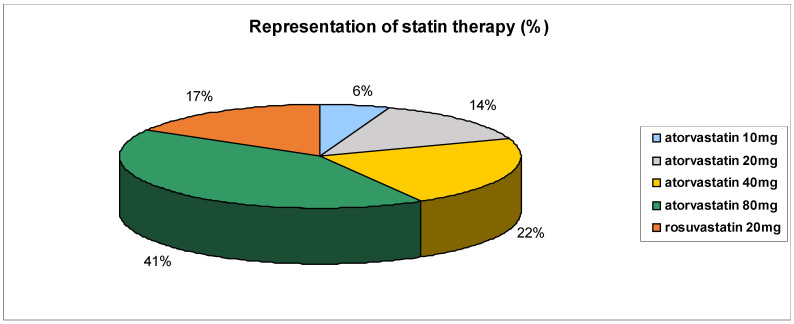
Representation of statin therapy (%) in n-3 PUFA group of patients.

In the n-3 PUFAs group, there were 56.5% (23) male and 43,5% (17) females, with a mean age of 60.5 years, range 31–81 years. MS was classified according to the definition of the IDF (2005) (International Diabetes Federation), with a discriminating factor being the presence of abdominal obesity [6]. Table 1 presents the patient medication at the start and at the end of the trial in the n-3 PUFAs group of patients, and Figure 1 presents the prevalence of the presence of International Diabetic Federation (IDF) criteria for MS. The main components include diabetes mellitus (DM), blood pressure (BP) triglycerides (TGs), high-density lipoprotein (HDL) and waist circumference (WC) As outlined in Table 1 statins were mostly used by 36 patients representing 90%. Figure 2 presents the use of individual statins at various doses in the n-3 PUFA group of patients.

Upon signing the informed consent form, all patients had a physical checkup, including blood pressure measurement, waist and hip circumference measurements, body height and body weight. Laboratory tests were performed, and each patient was evaluated using the EndoPAT device (Itamar Medical Ltd a subsidiary of ZOLL Medical Corporation, in the United States). At the beginning of the study, the patients initially received n-3 PUFAs (ZenixX Vital, Pleuran sro, Bratislava, Slovak Republic) at the dose of 2.4 g per day, divided into three daily doses of 800 mg. While a 1 g fish oil capsule, containing up to 200 mg of DHA and 300 mg of EPA, is recommended by the health service for preventive therapy, high-dose fish oil (up to >6 g/day) and concentrated omega-3 fatty acids (4 g/day) were used as triglyceride-lowering agents in patients with significant hypertriglyceridemia [7]. Based on the current clinical experience, this study used a medium dose.

All tests were performed before starting the study therapy and at the end of the subject period after three months (±1 week). The effect of treatment with n-3 PUFAs on ED was assessed on the following basis:

1. Selected laboratory markers and ED risk factors: glutathione-peroxidase (GPX), homocysteine (Hcy), lipoprotein (a) (Lp(a)), apo-lipoprotein B (ApoB);
2. Markers of arterial stiffness measured by augmentation index (AI) and the index of reactive hyperemia (RHI) measured as an endothelium function parameter were investigated using an EndoPAT 2000 device (Itamar Medical Ltd a subsidiary of ZOLL Medical Corporation, in the United States).

### 2.2. Laboratory Markers Evaluation

Of the assessed parameters, upon sample collection, the Lp(a), ApoB and Hcy were processed by a local laboratory. Specific ED markers such as GPX were sent in vials with heparin to the University Science Park-MEDIPARK, Pavol Jozef Safarik University, Kosice, Slovakia, where centrifuged plasma was frozen (−80 °C) until individual tests were performed. Upon thawing, a volume of 50 uL of heparinized blood was added to 2 mL of dilution solution. Upon the measurement of Hb concentrations in the resulting hemolysate, the glutathione peroxidase (GPX) activity was determined using a RANSEL kit (RANDOX, Dublin, Ireland), using the automatic analyzer Daytona. GPX activity in control samples was also determined with each measurement series for comparison.

### 2.3. Assessment of Endothelial Dysfunction Using EndoPAT 2000

**Figure 3:**
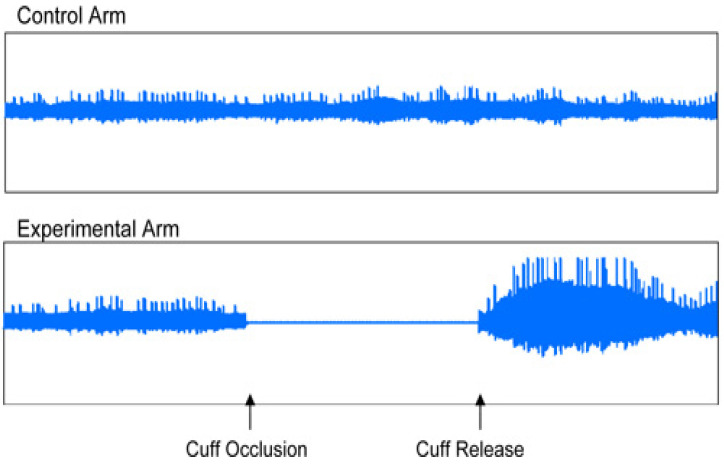
Original traces showing examples of normal endothelial function (vasodilation).

**Figure 4:**
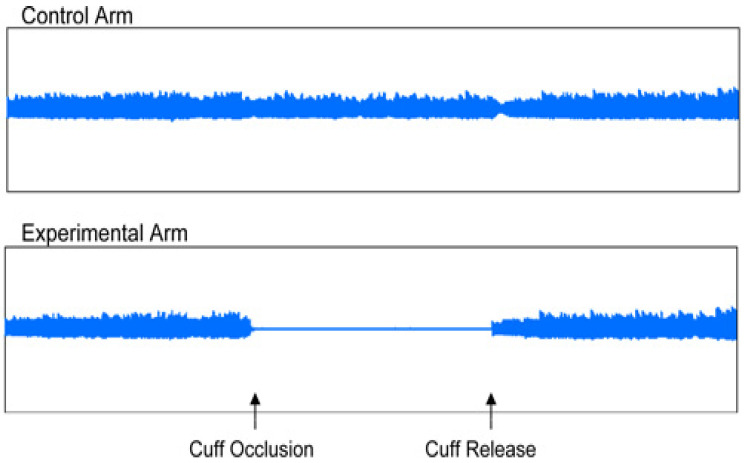
Original traces showing examples of endothelial dysfunction.

The EndoPAT 2000 was designed as a simple method for the non-invasive measurement of ED. The method involved the measurement of the changes of vascular tone in the peripheral vascular bed (PAT—peripheral arterial tone), using plethysmography, and the measurement was performed independent of the investigator. The EndoPAT assessment involved vasodilation–hyperemia following the previous application of a tourniquet on the extremity. The EndoPAT measured the signal from the vascular bed of the entire finger, including the microcirculation. The test enabled a differentiation between systemic and endothelial-induced vaso-reactivity. Moreover, the measurement was performed simultaneously on both upper extremities, and the resulting curves were compared. The assessment needed to be performed in a separate, quiet and warm room (Figure 3 and Figure 4).

Patient preparation before the assessment was required. In this study, each patient fasted for at least 4 h. They were asked to avoid nicotine, vitamins or any other medication for 8 h, as these would affect the vascular tone [8]. During the test, usually taking about 20 min, each patient was placed in a supine or semi-sitting position, most commonly with closed eyes, no talking and no movement of the upper extremities to avoid interference with the electrode signals. The sensing electrodes were placed on both index fingers and inflated, and subsequently, a signal check was carried out. The signal was recorded by the computer and observed by the investigator in the form of 2 curves throughout the test. During the first 5 min, the upper extremity was left without occlusion, and afterwards, the cuff was inflated using a pressure gauge to a pressure of at least 200 mm Hg for at least 5 min. Thereafter, the cuff was deflated, and the signal after occlusion was observed for at least an additional five minutes. The outcome of the test was automatically assessed employing the system software, using two parameters: reactive hyperemia index (RHI) and augmentation index (AI) [9].

### 2.4. Statistical Analysis

This study employed basic description statistics for continuous variables to describe the population. These included number, mean value, standard deviation, minimum and maximum values and a 95% confidence interval for mean values. For categorical values, absolute and relative frequency were used. To compare the mean values of continuous parameters for two or more groups, scatter analysis was used. The Chi-square test was used to compare categorical variables. A paired *t*-test was used to determine the effect of treatment, comparing the parameter values at the beginning and at the end of the therapy. When testing hypotheses, this study considered significance as a value of *p* < 0.05. The study was approved by the ethics committee from Pavol Jozef Safarik University in Kosice, Slovakia.

## 3. Results

The results of the study revealed that supplementation with n-3 PUFAs in MS significantly decreased ApoB (0.94 ± 0.36 vs. 1.13 ± 0.35, *p* = 0.001) and homocysteine (13.78 ± 3.05 vs. 19.3 ± 5.29, *p* = 0.001) and, concurrently, increased GPX (41.65 ± 8.90 vs. 45.20 ± 8.01, *p* = 0.001). On the other hand, Lp(a) did not change during this short pilot trial (0.30 ± 0.29 vs. 0.29 ± 0.33, *p* = NS) (Table 2). Among all the observed subjects, three patients (7.5%) failed to come to the follow-up visit after 3 months, due to voluntary discontinuation of the study treatment in the active group of patients. Moreover, no significant changes in laboratory biomarkers in the placebo group of patients were observed in this study.

In this study, very interesting results were observed upon testing the ED using the EndoPAT 2000. Of the 40 patients employed in this study, 37 of them displayed statistically significant differences in reactive hyperemia index (RHI), which is a marker for endothelial function, and augmentation index (AI), indicating arterial stiffness. The RHI increased from baseline values of 1.62 ± 0.42 to 1.96 ± 0.62 after treatment (*p* = 0.005), while the AI decreased from 14.66 ± 19.55 to 9.21 ± 15.64 (*p* = 0.002) after three months of treatment. No significant change or improvement was visible in the placebo group of patients during the trial (Table 3). Statistical significance was observed even between the groups of patients during the trial (Table 4).

## 4. Discussion

This pilot study employed a total of 40 n-3 PUFA-treated and 40 placebo patients in a short trial. According to the literature, initial small trials require about 20–80 subjects, compared to phase 2 trials which require 100–200 subjects. Instead of using the minimum of 20 subjects, it was felt that 40 patients would provide a convincing significant value comparing the two cohorts. In this study, the n-3 PUFA group consisted of 56.5% (23) male and 43.5% (17) females with a mean age of 60.5 years, range 31–81 years. Interestingly, many of these patients were working age in life.

Based not only on evidence-based medicine data but also on this current pilot trial, it is possible to conclude that n-3 PUFA represents a benefit for MS patients due to its lipid-lowering effect through its positive effect on atherogenic dyslipidemia. This study has also demonstrated that supplementation with n-3 PUFAs can lead to an improvement in ED in MS patients compared to a placebo group. Therefore, it is tempting to suggest that n-3 PUFA is exerting a protective beneficial effect on the atherogenic process in the blood vessels of the body. These findings suggest that there is a potential beneficial use for n-3 PUFAs in a comprehensive treatment of MS, especially since ED represents the basic common denominator in the pathogenesis of all components of MS.

ED occurs in the early stages of atherosclerosis, where disorders in the reactivity of blood vessels precede structural changes in the vascular wall, resulting from the joint action of all atherogenic and athero-protective factors. Most of the known risk factors of atherosclerosis, such as dyslipidemia, hypertension, diabetes, smoking, age, inactivity and menopause are all associated with ED. In the past 10 years, attention has been focused on additional important risk factors of atherosclerosis and ED. These include ApoB, ApoA, triglycerides, triglyceride-rich lipoproteins, small dense LDL particles, oxidized LDL, oxidized LDL antibodies, Lp(a), Hcy and C-reactive protein (CRP) measured using a high-sensitivity method (hsCRP) [9]. As such, it is important to discuss the risk factors and markers of ED observed in the current study.

The measurement of ApoB in MS patients is of paramount importance. These patients sometimes have normal levels, but their LDL consists of the population of so-called “small dense LDL particles”, with very high atherogenic potential. These small dense LDLD particles contain a greater percentage of ApoB, and the measurement of ApoB helps to better estimate the proportion of this high-risk population of LDL. The AMORIS study (Apolipoprotein Mortality Risk Study), as well as the large international study, INTERHEART, have shown that apoprotein B levels, as well as the apoB/apoA-I, ratio can significantly improve the estimate of cardiovascular risk [10]. In the current study, the data have shown that the supplementation of n-3 PUFAs in patients with MS can lead to a significant decrease of ApoB. Similar results over a period of 12 weeks have been shown also by the multicenter randomized study, MARINE [11]. Nevertheless, it must be admitted that only EPA (eicosatetraenoic acid) was supplemented in this study, as compared to our study, where a combination of EPA+DHA (deoxyhexose acid) was used, with EPA at a dose of at least 155 g and DHA at least at 520 g).

Another risk factor of ED is hyperhomo-cysteinemia (Hcy), even though its causal relation to CV risk is subject to controversy and discussion. Nevertheless, large, randomized studies such as NORVIT and HOPE-2 SEARCH failed to show that a reduction in Hcy would lead to a decrease in CV risk [12], like the multicenter, randomized, placebo-controlled, double-blind “SU.FOL.OM3” study.

In a study by Blacher et al. [13] they demonstrated that folic acid (group B vitamins) can elicit a significant decrease in Hcy following treatment with n-3 PUFAs, but surprisingly, this has not resulted in reduced CV morbidity. In the current study, following the supplementation with n-3 PUFAs, the results show a significant decrease in Hcy levels. However, to demonstrate the effect of CV morbidity, a longer observation is needed with a larger patient population, as well as employing a higher dosage of n-3 PUFAs than that used in SU.FOL.OM3. In turn, this could, therefore, be the objective of future clinical studies. However, an interesting finding involves the fact that Hcy, probably through increased vascular oxidative stress and atherothrombosis, can partially suppress the expression go GPX-1 gene, located at the 3p21.3 chromosome. Among many factors that contribute to the risk of atherosclerosis in plasma, great emphasis is placed on GPX-3, a basic extracellular peroxidase, that plays a significant role in the modulation of oxidative stress. A lack of GPX-3 is associated with the reduced biological availability of nitric oxide and the increased activation of platelets [14,15]. In the current study, following treatment with n-3 PUFAs, the data have shown a significant increase in GPX, thus confirming the parallel antioxidant effect of n-3 PUFAs, which can significantly affect ED and the process of atherogenesis.

Another independent risk factor of development of CV disorders is the increased level of Lp(a), as shown by currently available meta-analyses of epidemiological studies [16]. Lp(a) increases the risk of stroke and death associated with vascular events in elderly males, independently of LDL cholesterol levels. A reduction in Lp(a) is the secondary priority, following a reduction in LDL cholesterol and total cholesterol [16]. In the patient population of the current study, n-3 PUFAs did not significantly decrease Lp(a), and enrolling plasma levels did not reach significant values at all.

ED plays an important role in long COVID syndrome as reported by recent papers, where COVID-19 can impair ED directly either as a viral effect or via cytokines’ inflammatory response on endothelial cells to reduced nitric oxide bioavailability [17]. More common in patients with non-respiratory symptoms, long COVID-19 symptoms could be persistent due to ED, and thus, better care for the patients could be offered by n-3 PUFA supplementation. These conclusions need to be confirmed by another pilot, placebo-controlled trial.

In addition to the laboratory assessment of ED, the non-invasive measurement of ED using the EndoPAT 2000 is currently coming to the center of attention. This method involves the measurement of changes in vascular tone in the peripheral vascular bed (PAT—peripheral arterial tone) using plethysmography. The common principle for the EndoPAT 2000 and flow-mediated dilation (FMD) is vasodilation–hyperemia, following the previous occlusion of the extremity. A significant advantage compared to FMD is the fact that the EndoPAT 2000 is also a measure for arterial stiffness. Arterial stiffness is increased in various pathological conditions, such as ischemic heart disease, MS, chronic kidney disease and others [1]. The current study assessed endothelial function using the EndoPAT 2000, based on the values of two parameters, RHI and AI. There are no official reference values available for RHI, but in general, RHI values under 1.67 are classified as ED, while the higher values of RHI are considered normal or represent an improvement in endothelial function. The normal range of AI is between −30% and −10%, the limit value of AI is between −10% and 10%, and abnormal values of AI are above 10% [18,19]. This method was used to demonstrate the improvement in the parameters of ED and arterial stiffness in the MS patients in our study after 3 months of n-3 PUFA supplementation vs. the placebo group of patients.

The results of this study have clearly demonstrated that n-3 PUFA supplementation can exert significant beneficial effects in reducing and even preventing ED in susceptible MS patients. However, it is worth noting that ED is due to several risk factors, including hyperglycemia, overweight, obesity and increased waist circumference, high blood pressure and chronic stress, smoking, excessive alcohol consumption, inactivity or a sedentary lifestyle, elevated triglycerides and cholesterol, a diet rich in fats and sugar, a reduced fiber, vegetables and micronutrients intake normally found in Mediterranean diets and others [20]. In the present study, no questionnaire was employed to retrieve data about lifestyle changes, including inactivity, diet and others, from the two cohorts of patients who participated in this study. The question which now arises is: are the beneficial effects on ED due to the supplementation of n3-PUFAs or lifestyle changes or both? Since the placebo had no beneficial effect on ED, then it is tempting to suggest that n-3 PUFA is indeed reducing ED. Further experiments are required to distinguish between these two parameters, employing other groups of cohorts who have had no lifestyle changes and those who have had lifestyle changes. It is also noteworthy that these different risk factors can work synergistically to induce MS. As such, for n-3 PUFA supplements to have a far better beneficial effect in the treatment of MS, then susceptible patients must change their lifestyle habits, combined with an element of psychological intervention to adhere to the changes [21].

It is noteworthy that overweight and severe obesity are major recognizable risk factors globally among old and young subjects for the development of MS and several other medical conditions. Some of these disorders include type 2 diabetes mellitus (T2DM), hypertension, cardiovascular diseases (CVDs), hyperuricemia, inflammatory bowel disease, certain type of cancers, viral diseases, menstrual irregularities, asthma, osteoarthritis, chronic back pain, obstructive sleep apnea, non-alcoholic fatty liver disease, gall bladder diseases and others [22,23]. Obesity seems to be the trigger in initiating and developing coronary artery disease, heart failure, cardiac arrhythmias and strokes, which are responsible for most deaths globally [24,25]. Like the current study, several other studies have shown the beneficial actions of n-3 PUFAs in cardio protection, including hypertension, myocardial infarction, arrhythmias, strokes and sudden cardiac death [26,27]. Like obesity, diabetes is a major metabolic disorder that can lead to several long-term complications including cardiomyopathy and sudden cardiac death [28]. Several studies have demonstrated that n-3 PUFA supplements can control blood sugar levels and lipid profiles in patients with T2DM, thereby preventing diabetes-induced long-term complications [29].

### Limitations of the Study

The study was limited by the low number of subjects, affecting the results of the effect on ED. Another limitation is the duration of the observation period; a 3-month observation is not necessarily sufficient to assess the change in endothelial function. Additionally, it needed to be explained that the present study did not involve a homogenous population of subjects (statins treatment at various doses, monotherapy vs. combination therapy and the lengths of dyslipidemia treatment before the pilot trial starts. All this mentioned above could possibly affect the results due to known statins pleiotropic effects. Another source of possible errors involved the assessment using the highly sensitive device, EndoPAT 2000, despite our effort to ensure the strict observation of the requirements and procedures.

## 5. Conclusions

In this three-month observational study, the supplementation of n-3 PUFAs was able to improve ED in MS patients, as assessed by two selected markers, employing two different methods (laboratory and plethysmography measurements using the EndoPAT 2000). n-3 PUFA supplementation (2.4 g per day) was associated with a significant reduction in ApoB and Hcy and significant increase in the GPX antioxidant enzyme. Simultaneously, there was a significant improvement in arterial stiffness, as was assessed by a marked reduction in AI and an improvement in ED, accompanied by significant increase in RHI. This study did not observe positive changes in the placebo group of patients.

It is concluded that the effect on individual components of MS, based on the possible positive effect of the supplementation of n-3 PUFAs on ED, appears to be one of the promising and potential clinical alternatives available to modify the atherogenic process at a level that should be investigated by future clinical studies.

## Figures and Tables

**Table 1:** Medication in n-3 PUFA group of evaluated patients.

Medications	N. of Patients at Start	N. of Patients at End	*p*	%
ACEi	25	22	NS	62.50
Beta-blocker	32	32	NS	80.00
Calcium channel blocker	14	14	NS	35.00
Sartan–AT1 Blockers	12	12	NS	30.00
Alfa-blocker	12	12	NS	30.00
Diuretics	30	28	NS	75.00
Acetylsalicylic acid	15	15	NS	37.00
Trimetazidin	18	18	NS	45.00
Insulin	6	6	NS	15.00
Oral antidiabetics	12	12	NS	30.00
Nitrates	10	10	NS	25.00
Clopidogrel	7	7	NS	17.00
Allopurinol	8	8	NS	20.00
Proton pump inhibitors	2	2	NS	5.00
Warfarin	3	3	NS	7.50
Dabigatran	1	1	NS	2.50
Rivaroxaban	2	2	NS	5.00
Ivabradine	4	4	NS	10.00
Propaphenone	1	1	NS	2.50
Amiodarone	1	1	NS	2.50
Digoxin	2	2	NS	5.00
L-thyroxine	3	3	NS	7.50
Prasugrel	1	1	NS	2.50
Statins	36	34	NS	90.00
Fibrat	8	8	NS	20.00
Ezetimibe	6	6	NS	15.00

**Table 2:** Comparison of selected laboratory markers and risk factors of endothelial dysfunction before and after 3-month treatment with n-3 PUFA. Using paired *t*-test, data are mean ± SD; n = 37; * *p* < 0.001.

Lab Markers	Numbers	Before Treatment	After Treatment	*p*
Lp(a)	37	0.30 ± 0.29	0.29 ± 0.33	NS
ApoB	37	1.13 ± 0.35	0.94 ± 0.36	0.001 *
HCy	37	19.31 ± 5.29	13.78 ± 3.05	0.001 *
GPX	37	41.65 ± 8.90	45.20 ± 8.01	0.001 *

NS = not significant, GPX = glutathione peroxidase; HCy = homocysteine; Lp(a) = lipoprotein; ApoB = apo-lipoprotein B.

**Table 3:** Comparison of results of EndoPAT 2000 before and after 3 months of supplementation with n-3 PUFA vs. placebo group. Using paired *t*-test, data represent mean ± SD; n = 37; * *p* < 0.005.

n-3 PUFA Group	N	Before Treatment	After Treatment	*p*
RHI	37	1.62 ± 0.42	1.96 ± 0.62	0.005 *
AI	37	14.66 ± 19.55	9.21 ± 15.64	0.003 *
Placebo group				
RHI	40	1.51 ± 0.38	1.51± 0.38	NS
AI	40	16.46 ± 17.43	18.89 ± 13.49	NS

RHI—reactive hyperemia index; AI—augmentation index.

**Table 4:** Comparison of results of EndoPAT 2000 before and after 3-month supplementation with n-3 PUFA vs. placebo group (between groups). Using paired t-test, data represent mean ± SD; n = 37; *p < 0.001.

	Before Treatment n-3 PUFA	Before Treatment Placebo	*p*	After Treatment n-3 PUFA	After Treatment Palcebo	*p*
RHI	1.62 ±0.42	1.51± 0.38	NS	1.96 ± 0.62	1.58 ± 0.42	0.001 *
AI	14.66 ± 19.55	16.46 ±17.43	NS	9.21 ± 15.64	18.89 ± 13.49	0.001 *

RHI—reactive hyperemia index, AI—augmentation index.

## Data Availability

Data is unavailable due to privacy or ethical restrictions.

## References

[1] Bang H.O., Dyerberg J., Nielsen A. (1971). Plasma lipid and lipoprotein pattern in Greenlandic west-coast Eskimos. Lancet.

[2] Burr M., Gilbert J., Holliday R., Elwood P., Fehily A., Rogers S., Sweetnam P., Deadman N. (1989). Effects of changes in fat, fish, and fibre intakes on death and myocardial reinfarction: Diet and Reinfarction Trial (DART). Lancet.

[3] GISSI-Prevenzione Investigators (1999). Dietary supplementation with n-3 polyunsaturated fatty acids and vitamin E after myocardial infarction: Results of the GISSI-Prevenzione trial. Lancet.

[4] Von Schacky C., Angerer P., Kothny W., Theisen K., Mudra H. (1997). Study on Prevention of Coronary Atherosclerosis with Marine Omega-3 fatty acids (SCIMO)—First results of a randomized double- blind study. Can. J. Cardiol..

[5] Mori T.A. (2014). Dietary n-3 PUFA and CVD—A review of the evidence. Proc. Nutr. Soc..

[6] Alberti K.G.M.M., Zimmet P.Z., Shaw J.E. (2005). The metabolic syndrome-a new world-wide definition from the International Diabetes Federation consensus. Lancet.

[7] Asztalos I.B., Gleason J.A., Sever S., Gedik R., Asztalos B.F., Horvath K.V., Dansinger M.L., Lamon-Fava S., Schaefer E.J. (2016). Effects of eicosatetraenoic acid and docosahexaenoic acid on cardiovascular disease risk factors: A randomized clinical trial. Metab. Clin. Exp..

[8] Axtell A.L., Gomari F.A., Cooke J.P. (2010). Assessing Endothelial Vasodilator Function with the Endo-PAT 2000. J. Vis. Exp..

[9] Ganz P., Vita J.A. (2003). Testing endothelial vasomotor function. Nitric oxide, a multipotent molecule. Circulation.

[10] Yusuf S., Hawken S., Ounpuu S., Dans T., Avezum A., Lanas F., McQueen M., Budaj A., Pais P., Varigos J. (2004). Effect of potentially modifiable risk factors associated with myocardial infarction in 52 countries (the INTERHEART Study): Case control study. Lancet.

[11] Bays H.E., Ballantyne C.M., Kastelein J.J., Isaacsohn J.L., Braeckman R.A., Soni P.N. (2011). Eicosatetraenoic acid ethyl ester (AMR101) therapy in patients with very high triglyceride levels (from the multi-center, placebo-controlled, randomized, double-blind, 12-week study with an open-label extension [MARINE] trial). Am. J. Cardiol..

[12] Mori T.A. (2014). Omega-3 fatty acids and cardiovascular disease: Epidemiology and effects on cardiometabolic risk factors. Food Funct..

[13] Blacher J., Czernichow S., Horrellou M.H., Conad J., David P., Chadefaux-Vekemans B., Ankria A., Galan P., Hercberg S., Ducimetière P. (2005). Homocysteine, folic acid, group B vitamins and cardiovascular risk. Arch. Mal. Coeur Vaiss..

[14] Blankenberg S., Rupprecht H.J., Bickel C., Torzewski M., Hafner G., Tiret L., Smieja M., Cambien F., Meyer J., Lackner K.J. (2003). Glutathione peroxidase 1 activity and cardiovascular events in patients with coronary artery disease. N. Engl. J. Med..

[15] Prabhakar R., Morokuma K., Musaev D.G. (2006). Peroxynitrite reductase activity of seleno-protein glutathione peroxidase: A computational study. Biochemistry.

[16] Riche D.M., East H.E., Priest H.M. (2008). Practical management of dyslipidemia with elevated lipoprotein (a). J. Am. Pharm. Assoc..

[17] Varga Z., Flammer A.J., Steiger P., Haberecker M., Andermatt R., Zinkernagel A.S., Mehra M.R., Schuepbach R.A., Ruschitzka F., Moch H. (2020). Endothelial cell infection and endotheliitis in COVID-19. Lancet.

[18] Nichols W.W., Singh B.M. (2002). Augmentation index as a measure of peripheral vascular disease state. Curr. Opin. Cardiol..

[19] Jedlickova L., Merkovska L., Jackova Stovkova L., Janicko M., Fedacko J., Novakova B., Chmelarova A., Majernik J., Pella D. (2015). Effect of Ivabradine on Endothelial Function in Patients with Stabile Angina Pectoris: Assessment with EndoPAT 2000 Device. Adv. Ther..

[20] Kim L.M., Chung J., Kim K.J., Seo W.W., Jeon K.H., Cho I., Park J.J., Suh J., Lim S.-Y., Choi S. (2021). Lifestyle modification in the management of metabolic syndrome: Statement from Korean Society of Cardio-Metabolic Syndrome (KSCMS). Korean Circ. J..

[21] Martinus R., Corban R., Wackerhage H., Atkins S., Singh J. (2006). Effect of psychological intervention on exercise adherence in Type 2 diabetic subjects. Ann. N. Y. Acad. Sci..

[22] Pi-Sunyer F.X. (1999). Comorbidities of overweight and obesity: Current evidence and research issues. Med. Sci. Sports Exerc..

[23] Smail M.M., Singh J., Ismail A.M., Cummings E., Hanoman C., Rupee S., Rupee K., Adeghate E., Tapia P.S., Dhalla N.S., Ramjiawan P. (2020). Cellular and biochemical mechanisms driving the susceptibility of obese subjects to COVID-19 infection. Cellular and Biochemical Mechanism of Obesity: A Series of Advances in Biochemistry in Health and Diseases.

[24] Sultan A., Singh J., Howarth F.C., Tapia P.S., Dhalla N.S., Ramjiawan P. (2022). Cellular and Molecular Effects of Obesity on the Heart. Cellular and Biochemical Mechanism of Obesity: A Series of Advances in Biochemistry in Health and Diseases.

[25] Rea T.D., Heckbert S.R., Kaplan R.C., Psaty B.M., Smith N.L., Lemaitre R.N., Lin D. (2001). Body mass index and the risk of recurrent coronary events following acute myocardial infarction. Am. J. Cardiol..

[26] Abete I., Goyenechea E., Zulet M.A., Martinez J.A. (2011). Obesity and metabolic syndrome: Potential benefit from specific nutritional components. Nutr. Metab. Cardiovasc. Dis..

[27] Cebrian S.L., Costa A.G.V., Navas-Carretero S., Zabala M., Martinez J.A., Moreno-Aliaga M.J. (2013). Role of omega fatty acids in obesity, metabolic syndrome, and cardiovascular diseases: A review of the evidence. J. Physiol. Beachem..

[28] Rupee S., Rupee K., Singh R.B., Hanoman C., Smail M., Singh J. (2022). Diabetes-induced chronic heart failure is due to defects in calcium transporting and regulatory contractile proteins: Cellular and molecular evidence. Heart Fail. Rev..

[29] Bayram S.S., Kiziltan G. (2024). The role of omega-3 polysaturated fatty acids in diabetes mellitus management: A narrative review. Curr. Nutr. Rep..

